# Underwater cultural heritage and extreme events: Storm impacts under climate change

**DOI:** 10.1073/pnas.2523844123

**Published:** 2026-03-16

**Authors:** Luigi Germinario, Stuart J. McLelland, Claudio Mazzoli

**Affiliations:** ^a^Department of Geosciences, University of Padova, Padova 35131, Italy; ^b^Energy and Environment Institute, University of Hull, Hull HU6 7RX, United Kingdom

**Keywords:** underwater archaeological site, risk assessment, ocean currents, stone surface erosion, climate extreme

## Abstract

Extreme weather events cause severe ecological disruptions to the marine environment and socioeconomic impacts, while also endangering the evidences of human history resting underwater. Storms, in particular, generate high-intensity currents that lead to substantial material loss and textural alteration. We present a quantitative risk assessment of underwater cultural heritage exposed to storm events, considering climate change and the vulnerability of historical stone materials. We monitored the amount, rate, and patterns of stone erosion through innovative flume simulations and surface three-dimensional modeling, investigating its changes with time, current velocity, and properties of the materials and suspended sediments. The experimental results were combined with global models of storms and ocean currents under present and future climate, accounting for projected changes in storm intensity and frequency, climate scenarios, geographic settings, and seabed environments. Our findings show that even a single storm may result in irreversible damage to historical surfaces, erasing key morphological and textural features and compromising their legibility—especially in global hotspots such as tropical regions. In a future dominated by high greenhouse gas emissions and increasing extreme events, the vulnerability of archaeological stone could locally rise to more than double present levels. Extreme events and climate change pose major threats to the preservation of underwater cultural heritage and its historical, touristic, educational, and scientific values, requiring the development of long-term adaptation and protection strategies.

## Significance

Storms are among the most dangerous extreme events to the marine environment, causing violent currents that can damage underwater life, landscapes, and cultural heritage such as sunken ruins, wrecks, and archaeological remains. We studied the vulnerability of historical stone materials by simulating storm-driven currents in the laboratory and measuring their erosive effects caused by moving seabed sediments and water. We then applied present and future climate models to assess risk over time and space. Our results show that even a single storm can cause irreversible damage; risks are higher in regions affected by tropical cyclones and will increase under future climate change. These findings are crucial for predicting the future of our cultural heritage and guiding its protection.

Oceans and seas cover 71% of the Earth’s surface and are the guardians of an immense underwater cultural heritage, with millions of submerged settlements, wrecks, artifacts, and structures worldwide, carrying an extremely rich historical legacy (1, 2). Their preservation and protection depend on their resilience to environmental stresses, which may change due to anthropogenic climate change, involving ocean warming, acidification, deoxygenation, sea level rise, and increasing extreme weather events (3, 4). There has been significant debate on the ecological, socioeconomic, and health implications of climate change. On the sidelines, the risk to cultural heritage has also sparked a lively debate over the last 20 y (5–9), but mostly neglecting underwater sites and landscapes—at most, addressing just coastal areas, sea level rise, and coastal flooding and erosion. This flaw is evident in wide-ranging documents like the UNESCO Convention (10) and the recent IPCC reports [only briefly addressing shipwreck vulnerability (11, 12)]. Some studies contain speculations about the effects of stormy weather on the erosion and displacement of archaeological materials, or their aggravating corrosion and biofouling due to ocean warming, acidification, and salinity changes (13–17). However, the climate change impacts have never been quantified on a large scale, until the first risk assessment included in the recent pioneering study by Germinario et al. (18) on ocean acidification: Field and laboratory experiments coupled with climate models suggest limited vulnerability of underwater cultural heritage under preindustrial and present conditions, whereas high-emission future scenarios are associated with an exponential increase in stone decay due to marine dissolution processes. A further, smaller-scale example is provided by Ferrero-Martín et al. (19), who conducted a risk assessment of wave-induced hazards (decontextualization, scour, erosion) in a coastal setting, showing that the erosion risk is expected to increase locally under climate change, depending on sea level rise and wave energy flux; while closer in scope to the present study, their approach relies on wave climate models and does not consider extreme events, ocean currents, or laboratory validations.

Extreme weather events are among the most destructive environmental stressors, manifesting as temperature extremes, heavy precipitation, storms, floods, droughts, or wildfires. Storm events, in particular, can have significant impacts on the marine environment, generating storm surges, large waves, strong currents, and physical and biogeochemical changes, involving seawater temperature and salinity, plankton blooms, etc. (20, 21). A major contribution is provided by near-inertial mixed-layer and thermocline currents, generated by the transfer of wind-stress kinetic energy into the upper ocean during storm passage; the response, primarily controlled by storm characteristics (intensity, speed, size) and environmental conditions, is asymmetric and gradually decays over several days to weeks through wave dispersion and propagation (20, 22). Deep water may also experience disturbances, although much weaker and longer-lasting (23).

The mentioned hazards damage marine habitats and ecosystems, as well as fisheries, aquaculture, and infrastructure, and are set to worsen with global warming (12). In general, there is no consensus on consistent patterns of future changes of storm frequency, which is expected to either remain unchanged or decrease. However, global warming amplifies the available heat energy and storm thermodynamic potential (24). This results in often stronger winds, higher precipitation rates, larger proportion of violent events, and track shifts, with more severe tropical cyclones, extratropical cyclones, and even mesoscale hybrid tropical-like systems such as Mediterranean cyclones (medicanes) (25–31).

This article presents a quantitative risk assessment of underwater cultural heritage exposed to extreme weather events, simulating and predicting the effects of high-intensity ocean currents driven by storms. Stone vulnerability represents the main focus, considering the extraordinary historical significance of this natural material and its widespread usage in ancient buildings, harbors, roads, sculptures, tools, and ship cargoes now underwater. The amount, rate, and patterns of stone decay were monitored during innovative flume experiments, simulating fast water flows with high suspended sediment loads. Finally, the vulnerability in space and time of archaeological stone materials to natural storm events was modeled under present and future climate conditions.

## Surface Changes.

### Material loss.

**Fig. 1.**
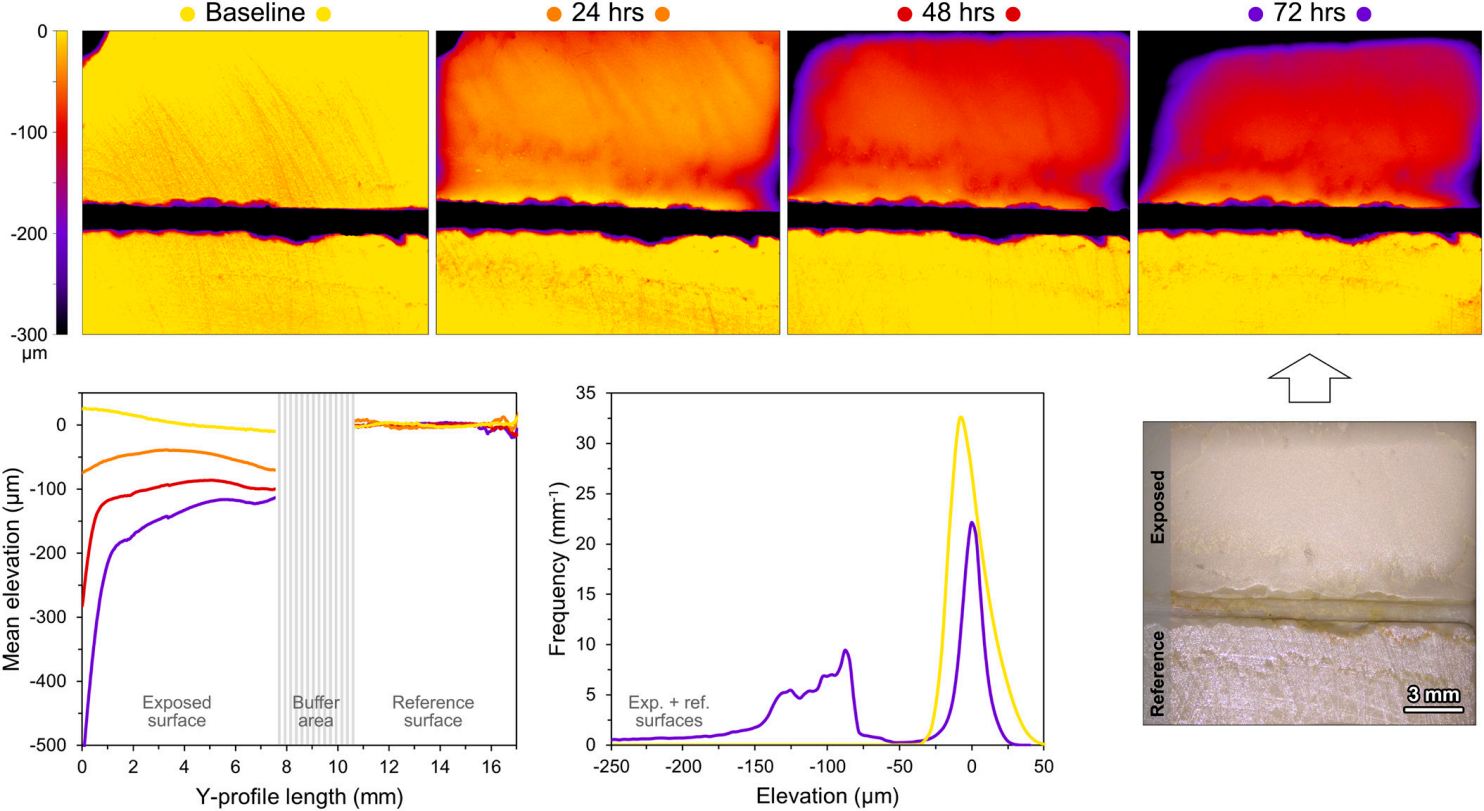
3D models showing the surface evolution of a specimen of compact limestone during the flume experiments with maximum flow velocity and coarse sand, with the corresponding topographic trends and elevation distributions.

**Fig. 2.**
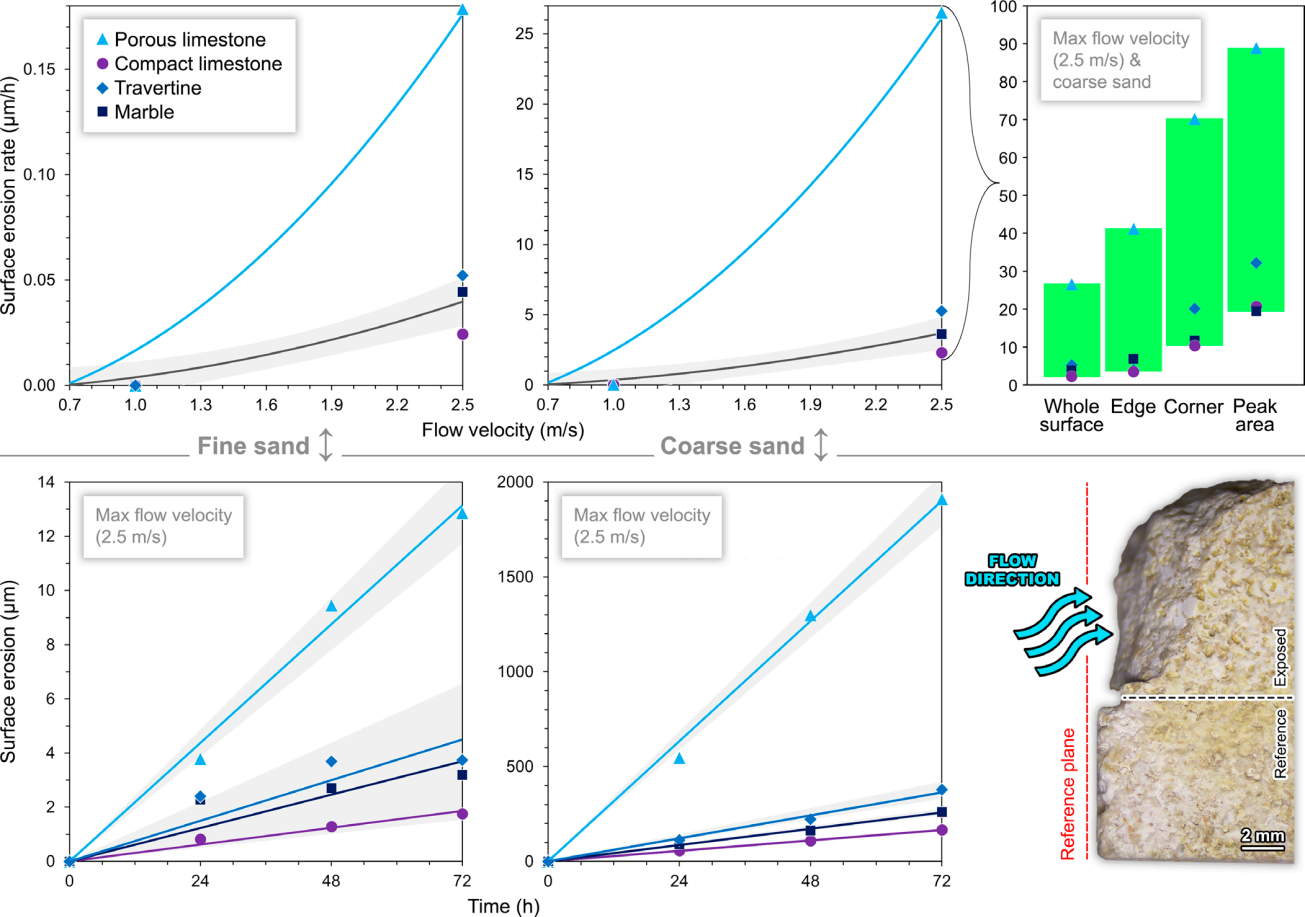
Stone surface erosion at different water current velocities with suspended fine and coarse sand; for the maximum flow velocity, additional data on the differential erosion and temporal trends are shown, together with the side view of a specimen of porous limestone at the end of the most extreme simulations (markers = experimental data points; curves = best-fit quadratic regression models with f(0) = 0, one for porous limestone, one for all the hard stones; lines = best-fit linear regression models; shaded areas = 95% confidence bands, with possible overlaps; full dataset and regression metrics in Dataset S1).

The most significant indicators of deterioration for the selected historical stone materials (*SI Appendix,* Fig. S1) are the amount and rate of surface material loss and disintegration due to the repeated dynamic abrasive action of impacting suspended sediments and water. All experimental data are enclosed in Dataset S1, with the main findings summarized in Figs. 1 and 2. With suspended fine sand, stone material loss is very limited, and measurable only with the 2.5 m/s water flow: After 72 h of experimentation, it totals 2 to 4 µm for compact limestone, marble, and travertine, and 13 µm for porous limestone. With suspended coarse sand, stone surface erosion still amounts to a few µm with the 1.0 m/s flow, but at 2.5 m/s it is discernible even to the naked eye, reaching 166 µm for compact limestone, 261 µm for marble, 379 µm for travertine, and 1,909 µm for porous limestone; these values correspond to an average erosion rate of 2 to 5 µm/h for all materials except for porous limestone, which records 27 µm/h. Hence, the combination of coarse sand and very high current velocity results in the most extreme decay. These experimental conditions also enable the identification of differential erosion patterns; disintegration is generally twice as rapid at the edges, and three to five times greater at the corners most exposed to the water flow (with an erosion rate of 10 to 70 µm/h).

In general, stone erosion rate follows a quadratic increase with current velocity, with measurable effects only above 0.7 m/s. The squared-X regression model is limited by the number of observations, but is still useful for reconstructing material decay in natural conditions of unsteady currents. The quadratic models capture most of the observed variability, i.e., 85 to 98%, with deviations between predicted and measured rates reflecting stone- and sediment-dependent differences. Moreover, material loss increases over time following a strongly linear trend, which typically explains at least 94% of data variability at high current velocities, with prediction errors often indicating low uncertainty (Dataset S1). This is consistent with the literature on the dynamics of stone wear during constant-rate abrasion processes (e.g., ref. 32).

### Textural alteration.

**Fig. 3.**
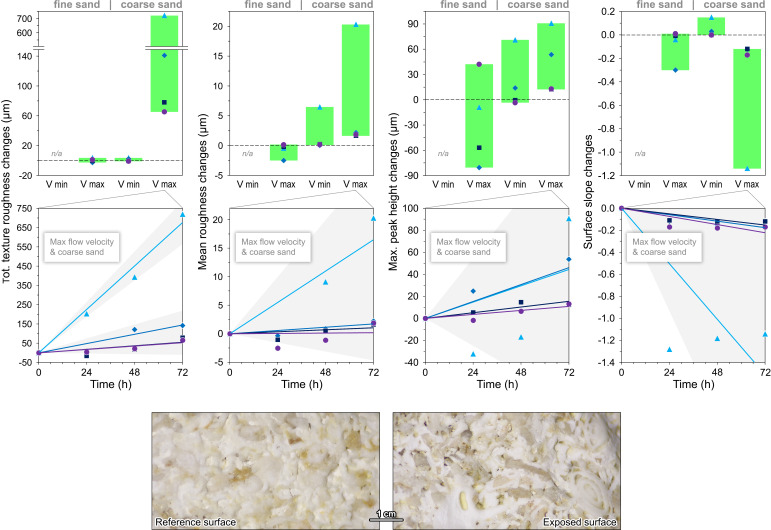
Stone textural changes at different water current velocities (v_min_ = 1.0 m/s; v_max_ = 2.5 m/s) with suspended fine and coarse sand, expressed by the deltas of representative morphometric parameters relative to the initial state (markers as in Fig. 2); for the maximum velocity and coarse sand, the temporal trends with their 95% confidence bands (shaded, with possible overlaps) are shown, together with photomicrographs of a specimen of porous limestone at the end of the experiments (full dataset and regression metrics in Dataset S1).

An alternate approach to interpret the stone surface changes is through the lens of textural alteration. The results of the morphometric analyses (Dataset S1) are summarized here by considering four representative parameters: total texture roughness (unfiltered mean roughness), mean roughness, maximum peak height, and surface slope (33). Fig. 3 depicts their evolution, cross-combining all experimental conditions. As with material loss, the greater the sediment grain size and the current velocity, the more pronounced the textural changes. These are usually negligible and variable in sign, except for the combination of coarse sand and highest flow velocity, which reveals clear trends of increasing roughness and decreasing surface slope, often linearly over time. The most extreme simulations also allow distinguishing more easily the behavior of different materials. In fact, after 72 h, total texture roughness increases by 65 µm for compact limestone, 78 µm for marble, 142 µm for travertine, and 721 µm for porous limestone. Mean roughness increases by 2 µm for all materials except for porous limestone (20 µm). Maximum peak height follows a similar pattern, but is very sensitive to topographic outliers. Finally, surface slope decreases by 0.1 on average (1.1 for porous limestone); this decrease, apparently inconsistent, is likely connected with the leveling of preexisting sharp-edged and steep microrelief (e.g., saw-cut marks) (34). Microscopic observations under multiple-angle illumination confirm that the surfaces of compact limestone, marble, and travertine lose reflectance and polish, and their edges become rounded (Figs. 1 and 2); in contrast, porous limestone is mainly affected by differential erosion, with bioclasts raising in relief above the surrounding weaker matrix and other micritic domains (Fig. 3).

### Environmental and material constraints.

These findings provide an indirect perspective on the characteristics of stone decay in the dynamic context of strong ocean currents, which is controlled by both environmental and material-related factors.

Flow velocity, availability of mobile sediments, and their grain size determine the quantity of particles that can be mobilized and their impact force: The higher the flow velocity and the finer the bed’s grain size, the greater the amount of material in suspension (35–37). An underwater stone artifact acts as an obstacle, which alters the flow pattern and results in a local change of current velocity, also depending on small-scale geometric features of the stone itself (hence, for this study, flow velocity was represented using the free-stream velocity upstream from the exposed samples). Therefore, the abrasion generated by the impact of grains can show small variations and be associated with differential erosion.

Deterioration rates and patterns are also dependent on the textural and technical properties of the materials investigated (*SI Appendix,* Fig. S1). Although mineralogical composition and hardness (~3 on the Mohs scale) are the same for all stones, their diverse textural features influence their mechanical strength and the distribution of shear and tensile microcracks resulting from abrasive stresses (38). Porous limestone—hereafter “soft stone”—is by far the softest and least dense material, characterized by weak intercrystalline bonds and the lowest durability (18); comparing the abrasion resistance of other rock types, porous limestone is closer to materials like volcanic tuff and shale. In contrast, compact limestone, marble, and travertine—hereafter “hard stones”—show performance more similar to volcanic rocks and sandstones, but poorer than granites and other hard plutonic rocks (38).

It is important to recall that underwater erosion of rocky substrates—whether man-made structures and artifacts or natural bedrocks and coral reefs—may be driven by physical, chemical, or biological processes. Physical processes, as noted above, arise from the transport of sediments in suspension or by saltation, damaging rock substrates by abrasion, plucking, cavitation, and scour; these mechanisms have been investigated especially in fluvial environments (39, 40). Chemical processes are associated with mineral dissolution, controlled by seawater chemistry (pH, CO_2_ content, etc.) and temperature, and exert especially severe impacts on carbonate substrates (41, 42). Finally, biological processes are mainly linked to the activity of marine organisms colonizing the substrate or its pores (43).

## Heritage Risk Assessment.

### Vulnerability to single storm events.

To formulate a risk assessment for underwater cultural heritage and quantify stone decay in natural settings, the experimental data were combined with the dynamic properties of typical storms (intensity and translation speed) and storm-driven currents (velocity and direction).

**Fig. 4.**
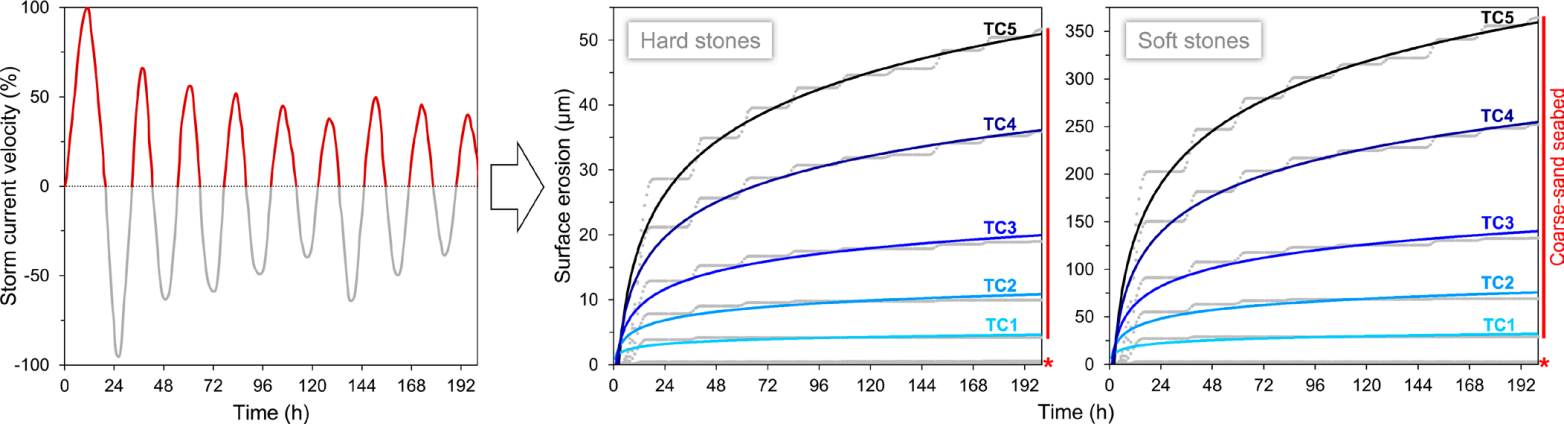
Reference normalized model of the single-axis bidirectional velocity component of storm-driven ocean currents [after (44)], filtered and rescaled for calibrating temporal surface erosion trends of hard and soft stones during a single storm event, for different seabed environments and storm categories (equivalent SSHWS TC/tropical cyclones and TS/tropical storms); the data best visible in the graphs are associated with tropical cyclones and coarse-sand seabed, whereas all erosion values linked to tropical storms and/or fine-sand seabeds plot very close to the x-axis (marked with a star).

Fig. 4 shows the bidirectional velocity profile of a representative upper-ocean current induced by wind stress from a tropical cyclone (normalized after 44). Currents peak during the forced stage (typically lasting half a day) with the storm passage, and weaken during the relaxation stage (5 to 10 d) (45). Current strength depends on storm intensity, translation speed, size, water column stratification, seabed topography, etc. (20), the most impactful parameter being wind. Current velocity in the ocean mixed layer roughly corresponds to 2 to 4% of wind speed (46, 47) and linearly increases along the Saffir–Simpson hurricane wind scale (SSHWS) (48); this ratio is modulated by storm asymmetry and inertial resonance, latitude and the Coriolis effect, stratification, and the relative contributions of ageostrophic and baroclinic currents. The strongest currents are generally located 50 to 100 km from the storm and can reach velocities of up to 2.5 m/s (46, 48).

In view of this, the reference velocity model was one-direction filtered and rescaled based on the SSHWS ranks and coupled with the experimental quadratic models of stone surface erosion; this data processing allowed calibration of stone decay trends for single storm events (Fig. 4 and Dataset S2). The first half-day of the storm is the most critical phase, with material loss reaching 50%, then rising to 90% within 2 to 6 d (depending on storm intensity). Fine-sand seabed environments are the least vulnerable and experience negligible stone erosion at the sub-μm scale, never exceeding 1 to 2 μm even after the most violent storms. With coarse-sand seabeds, the picture is different: Only SSHWS tropical storms cause negligible stone material loss, which increases 10-fold at the next level, i.e., category 1 tropical cyclones; the records during the strongest tropical cyclones (category 4 and 5) are 36 to 52 μm for hard stones and 252 to 364 μm for soft stones. In general, the erosion of soft stones is about 4 times higher with fine sand and 7 times higher with coarse sand.

### Vulnerability in space and time.

**Fig. 5.**
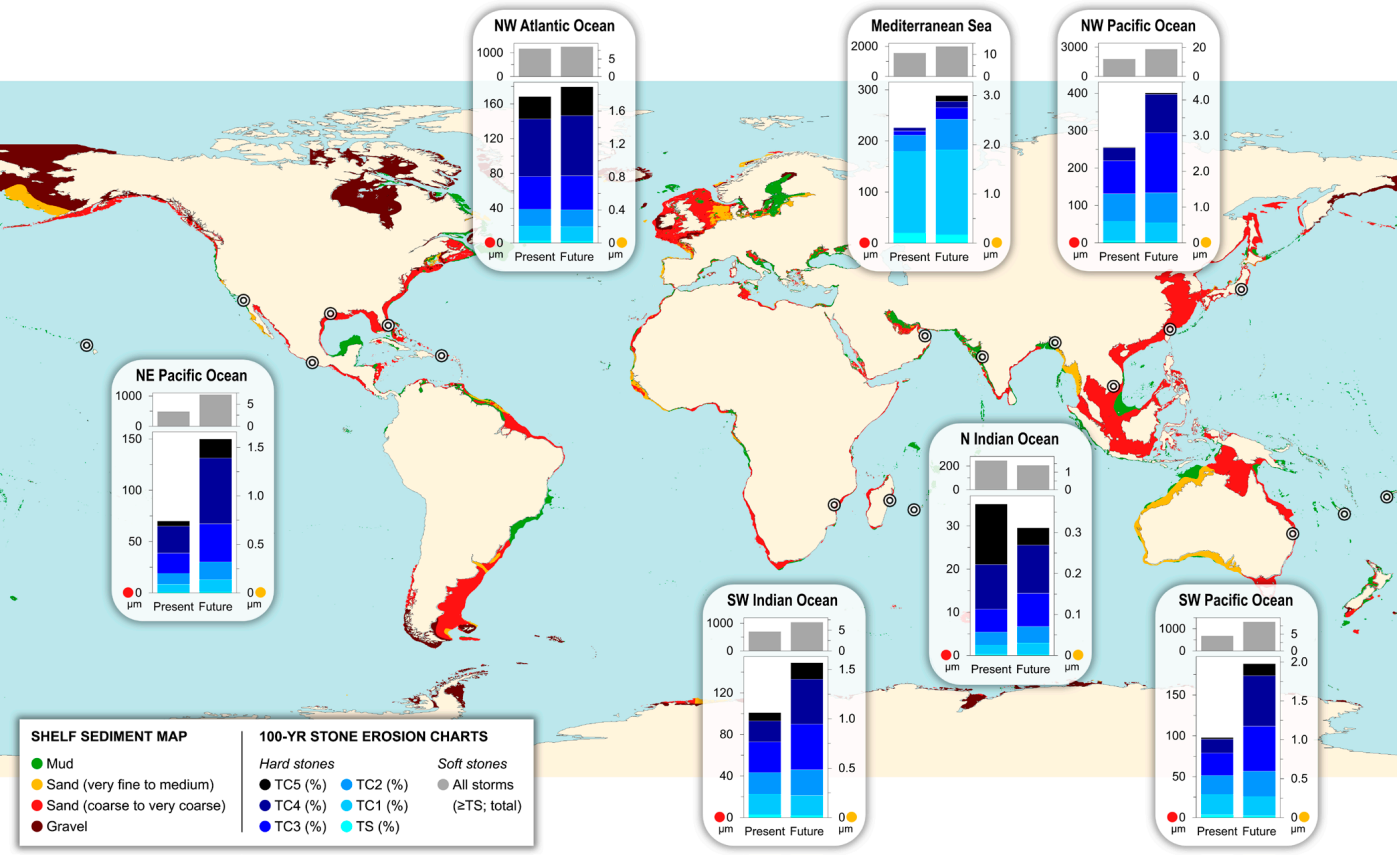
World seabed sediment map of continental shelf areas (max depth 200 m), with charts of underwater stone erosion caused by storm-driven currents modeled in 100 y for different basins. Each *Inset* indicates the total surface material loss in µm comparing the present and future ocean conditions and seabed environments dominated by coarse sand (left Y-axes) and fine sand (right Y-axes). The bottom charts refer to hard stones and include the total erosion together with the proportional contribution of different storm classes (categorized as equivalent SSHWS TC/tropical cyclones and TS/tropical storms). The top charts refer to soft stones and only show the total erosion. The bullseye symbols mark the coastal locations considered for averaging the return period data from each tropical basin (further details on the creation of the map and plots are in *Materials and Methods*).

Assessing the vulnerability of a site requires looking beyond the impact of single storm events to incorporate the recurrence and varying intensities of storm activity over time, particularly in regions identified as hotspots for extreme weather. Focusing on upper ocean/continental shelf environments worldwide, this risk assessment considered site-specific threats comparing present and future ocean conditions—i.e., accounting for the climate change impact on extreme weather events and the IPCC future scenarios of high greenhouse gas emissions. The resulting data are in Dataset S3. Fig. 5 displays the modeled stone surface erosion in underwater heritage sites over one century, targeting the coastal and shelf regions most affected by tropical cyclones and the Mediterranean basin, through relevant storm return periods (26, 30)—while excluding the minor contribution of the weaker and less destructive extratropical cyclones at mid-latitudes.

With regard to tropical cyclones, the region with the highest risk is by far the NW Pacific Ocean. Under present climate, the NW Atlantic, SW Indian, SW Pacific, NE Pacific, and N Indian follow in order. Combining their intensity and relative frequency, category 3 and 4 tropical cyclones yield the greatest proportional contribution to stone material loss (over 50% on average), whereas tropical storms have a negligible impact. Under future climate, the overall risk and the impact of the most violent storms increase almost everywhere, with stone material loss even doubling in the Pacific regions. For hard stone surfaces over coarse-sand seabeds, the regional average material loss over 100 y ranges from 35 µm in the N Indian basin up to 256 µm in the NW Pacific under present climate, and from 30 to 401 µm in the future scenario; on the other hand, fine-sand seabeds are associated with values around few µm. The modeled erosion of soft stones is significantly greater, reaching maximum regional averages of 1.8 and 2.8 mm under present and future climate scenarios, respectively. It is worth stressing once more that these are regional averages, and the local risk within the same region may increase significantly.

With regard to medicanes, their intensity is significantly lower. The western and central Mediterranean are the regions with the highest risk, but the model presented here overestimates stone damage, being based on return periods for the whole basin. There, equivalent SSHWS category 1 tropical cyclones (i.e., medicanes with intensity within the SSHWS category 1) account for the largest proportional contribution to stone material loss (over 70%), which is projected to decline slightly in the future, due to the increasing frequency and impact of stronger storm events. Hard stone surfaces are predicted to experience only a minimal increase in the 100-y material loss comparing present and future climate, i.e., from 226 to 289 µm over coarse-sand seabeds, and almost none over fine-sand seabeds. Again, the vulnerability of soft stones is higher and the maximum erosion values can be as great as 2 mm.

**Fig. 6.**
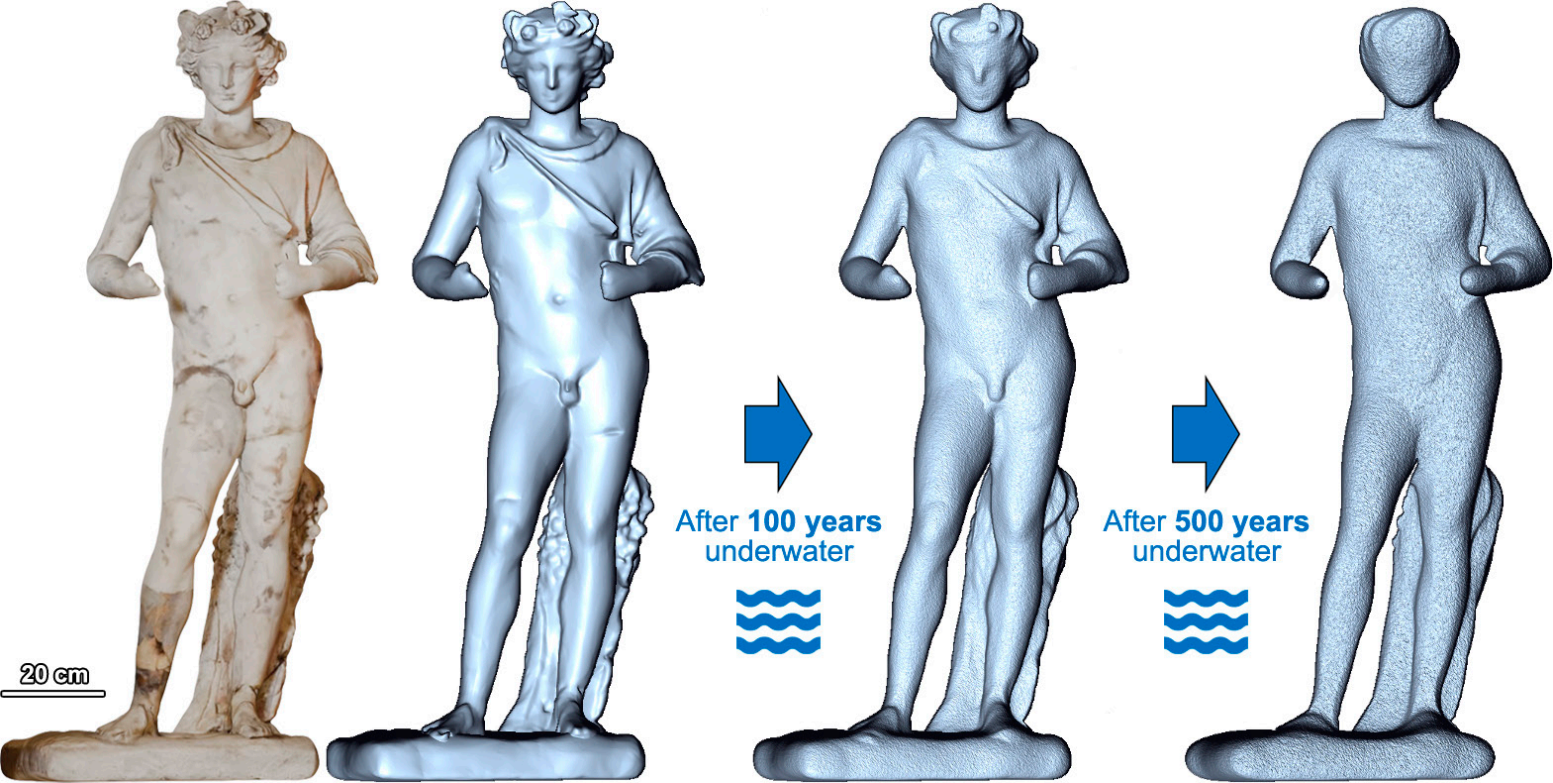
Textured and untextured 3D model of the 1st-century CE marble sculpture of Dionysus, formerly underwater in the sunken Roman city of Baia and currently in the Archaeological Museum of Campi Flegrei, Italy. The original mesh was digitally reshaped in Blender and Gwyddion, adjusting the elevation parameters based on the modeled stone erosion over coarse-sand seabeds, considering the global maxima (from the NW Pacific Ocean) and assuming a uniform surface material loss. Two predictions of underwater marble deterioration caused by storm-driven currents after 100 and 500 y are presented under the IPCC high-emission future scenario (original 3D model by 3D Research Srl, MUSAS project of Istituto Centrale per il Restauro, PON “Cultura e Sviluppo” FESR 2014-2020 funds).

To visualize the extent of damage that archaeological materials may undergo, Fig. 6 shows 3D simulations of future deterioration of a real marble sculpture underwater, considering the highest-risk location from the regional models above: Within 100 y, the artifact would lose its fine sculptural details, while within 500 y its morphological and textural features would largely disappear, resulting in a complete loss of legibility.

This risk assessment allows exploration of the implications of different storm intensities and frequencies, climate scenarios, material properties, geographic settings, and seabed environments, and is scalable according to different time frames and climate projections. Moreover, it can be readily tailored to the specific seabed characteristics of a heritage site; most of the locations considered here have sandy seabeds, and so do most shelf regions worldwide (Fig. 5), which aligns well with the experimental data. In contrast, it may be inferred that muddy seabeds pose no hazard, whereas gravelly seabeds may be associated with the highest risk; nonetheless, gravel typically requires extremely energetic hydrodynamic conditions to be mobilized and, thus, actual abrasion effects would be less frequent.

As final remarks, the limitations of this approach need to be highlighted.

The computed erosion trends are derived from simplified climate models that, by their nature, have limited accuracy, as they simulate rare, short-lived, local, and individual extreme events marked by stochastic variability; future projections are even more challenging due to the difficulty of capturing small-scale physical processes at high resolution (29). See further details in *Materials and Methods*.

Moreover, the extrapolation of laboratory data to natural settings does not fully consider the physical link between large-scale hydrodynamic forcings and small-scale processes, with stone erosion being controlled also by near-bed water velocity, turbulence, and sediment fluxes. In archaeological contexts, these relationships are established through numerical modeling of current- and wave-induced flow fields coupled with sediment transport formulations (49–52). These simulations require high-resolution parameterization of seabed morphology (bathymetry, roughness, etc.), sediment availability, and object geometry, as key prerequisites for resolving local flow acceleration, turbulence generation, and sediment mobilization (53–55). This especially applies to energetic conditions (56–59), where current-driven erosion may also act in tandem with storm surge, as temporary sea level rise and enhanced wave-induced orbital velocities increase seabed shear stress and scour (60) [short-lived extreme forcings can produce changes comparable to long-term background hydrodynamic conditions (61)]. The high computational efforts required by these hydrodynamic-sediment-erosion models limit their application typically to individual sites or high-risk locations (51). Therefore, this study adopts a complementary and conservative approach: The laboratory-derived stone decay laws can be treated as transferable constitutive relations, provided that relevant hydrodynamic boundary conditions are independently resolved; this allows moving from global-scale scenarios to screening-level risk assessments of local conditions.

Finally, this study does not consider explicitly other concurrent processes such as biofouling, scour, material displacement and scattering (51), burial and reexposure, some of which cannot be effectively simulated in the laboratory. These processes may act in synergy and amplify the damage associated with extreme events. For example, bioerosion can compromise the structural integrity of a wide range of substrates, both natural and man-made, which can be colonized by marine microborers (algae, fungi, and cyanobacteria), macroborers (sponges, bivalves, polychaetes, sipunculids, and echinoids), and grazers and scrapers (chitons, gastropods, echinoids, and fishes) (43, 62–65). At the same time, biocolonization can even shield the substrate from hydrodynamic erosion. Given these uncertainties, this study focuses on representing the physical response of bare stone surfaces and providing a baseline that can be modulated by biological factors in site-specific applications.

## Conclusions

Extreme weather events severely disrupt the marine environment and pose a major threat to underwater cultural heritage. Storms, in particular, generate high-intensity currents that can cause significant surface material loss and textural alteration of historical stone materials. A single storm event may result in erosion on the order of tens of µm, or even hundreds for the softest materials, especially in global hotspots such as tropical regions. Anthropogenic climate change, along with the projected increase in storm intensity on a global scale, is expected to exacerbate this trend. In a future dominated by high greenhouse gas emissions, the vulnerability of archaeological stone surfaces could rise up to double or more the present levels in the highest-risk areas (such as the Pacific Ocean); this scenario is not unlikely, as emissions have yet to peak and the impact of international policies and recent climate COPs appears insufficient.

This study presents a spatially and temporally resolved risk assessment that explores the implications of varying storm intensity and frequency, climate scenarios, material properties, geographic settings, and seabed environments; the broad-ranging quantitative indications on stone vulnerability can be tailored for higher-resolution investigations of specific sites. The findings highlight a clear challenge for the in-situ preservation of underwater cultural heritage that—although often hidden from view—represents an extraordinary historical legacy and carries significant touristic, educational, and scientific value. Therefore, there is an urgent need for long-term strategies and policies for adaptation and protection.

Finally, this approach opens avenues for future studies of other archaeological materials and interdisciplinary applications, including the assessment of storm impacts on coral reefs and marine rocky landscapes.

## Materials and Methods

### Stone Selection and Characterization.

This study focused on carbonate rocks for two reasons: their historical importance and widespread usage and the need to treat composition and mineral hardness as constants, while considering a range of different textures and technical properties typically observed in cultural heritage (66, 67). Following this direction, four representative materials were selected: marble (Carrara marble), travertine (Roman travertine), compact limestone (Istria stone), and porous limestone (Vicenza stone). Before the experiments, they were characterized with the following methods:

- Polarized-light microscopy on thin section, to carry out the petrographic classification;
- X-ray fluorescence (XRF), by a wavelength-dispersive spectrometer Panalytical Zetium, to analyze the bulk geochemical composition, with prior separate quantification of loss on ignition (LOI);
- Saturation and buoyancy weighing (68), to determine open porosity and bulk and matrix density.

These analyses and the relevant data, shown in *SI Appendix,* Fig. S1 together with information on the historical background, are shared with a previous study conducted on the same materials (18).

### Laboratory Simulations.

The laboratory simulations were conducted in an experimental flume facility (*SI Appendix,* Fig. S2). The working section of the flume channel is 0.5 m wide, up to 0.5 m deep, and 10 m long. It is equipped with a propeller pump system mounted in the outflow pipe for recirculating water and mobile sediment; five General Acoustic UltraLab water surface elevation gauges (0.18 mm resolution); a Nortek Vectrino mounted on a manual stage for measuring the average flow velocity at 40% of the flow depth; and an acoustic system mounted in the return flow pipe for continuously measuring the discharge. Before the experiments, the flume was tilted to 2.4% to achieve the maximum possible flow velocity without significant air entrainment, thereby ensuring a constant flow regime.

The arrangements for the experiments consisted of three steps:

- Sediment selection. Quartz-rich sands were used to mimic the seabed of shallow marine environments (inner continental shelves), which are typically dominated by siliciclastic terrigenous sediments, mostly in the sand range with minor gravel (69–71); shallow environments also feature the largest presence of underwater cultural heritage and are affected by stronger natural currents. Two grain sizes were selected: fine sand, with the same size distribution as common shelf sediments (72–75), and coarse sand, with a size distribution typical of higher-energy shelf settings. The grain size distribution was determined by a laser diffraction analyzer Malvern Mastersizer 3000 within the 0.01 to 3,500 µm range (*SI Appendix,* Fig. S3).
- Stone preparation. A set of 20 × 20 × 45 mm stone specimens was prepared, with their 20 × 20 mm faces set to be exposed to the water flow and divided by a groove into two areas: one directly exposed to the flow without any protection; the other shielded by a multilayered, seamless, and waterproof padding of self-amalgamating tape, providing an erosion-free reference surface (*SI Appendix,* Fig. S2).
- Flume setup. The flume was filled with 600 kg of sediment and with water to an average depth of 10 cm. Sediment and water were recirculated in order to redistribute the sediment throughout the working section and create a uniform, equilibrium bed (this procedure was followed twice, for the fine and coarse sand separately); the average sediment bed thickness upstream was 5 cm, with adjustments depending on the sediment type. Finally, each stone specimen was positioned and anchored on the sediment bed, with one edge resting on another adjacent specimen and the opposite edge free; the specimens were oriented with their shielded surfaces on the bottom, where sediment transport is slower.

Four simulations under different experimental conditions were conducted, cross-combining the two sediment grain sizes with two distinct average flow velocities, continuously monitored throughout every test via water surface elevation and discharge measurements: 1.0 m/s (corresponding to a discharge of 0.06 m^3^/s) and 2.5 m/s (0.13 m^3^/s). Each simulation lasted 72 h in total, and every 24 h, the stone specimens were temporarily removed, cleaned, oven dried at 70 °C, and subjected to the mid-term investigations, after which they were reinstalled, with a fresh set of specimens used for each simulation. During the experiments, the suspended sediment concentration was monitored and calculated from sediment samples collected upstream, in front of the stone specimens (the highest concentrations were recorded with the highest velocity flow: 119 ± 27 g/L for fine sand and 12 ± 8 g/L for coarse sand).

### Analysis of Stone Deterioration.

Stone deterioration caused by the physico-mechanical stresses associated with the flume currents was monitored and quantified in terms of surface erosion and textural alteration, as well as their evolution over time. The investigation approach was mainly based on the creation of high-resolution 3D models of the stone surfaces and their morphometric analysis, supported by stereomicroscopic observations under multiple-angle illumination.

The models were acquired with a noncontact 3D optical profilometer Nanovea Jr25, equipped with two optical pens providing a fixed vertical resolution of 3.4 and 41.0 nm within a z-measurement range of 1.4 and 24.0 mm, respectively; the lateral resolution (xy-step size) selected was 20 µm, with a scan speed between 2 and 20 mm/s (100 to 1,000 Hz). The choice of optical pen and scan speed was adjusted according to the stone surface characteristics (asperity, reflectivity, etc.). The topographic mapping was performed before the flume experiments, at intermediate steps, and at the end, analyzing the entire area of the specimens (20 × 20 mm), in order to maximize statistical representativeness, including both the exposed stone and the shielded reference surface. The processing and analysis of the 3D models was done with the software Gwyddion v2.67 (Czech Metrology Institute) following a five-step procedure.

- Image framing and correction, and Laplace’s interpolation of minor outliers.
- Leveling and rescaling, subtracting the least-squares mean plane and setting the reference surface to zero.
- Microroughness filtering, applying a conservative denoise filter and a Gaussian smoothing filter.
- Frequency filtering, separating the short-wavelength roughness component from the long-wavelength waviness component with a cut-off (nesting index) of 2.5 mm.
- Areal statistical analysis, computing the textural parameters of the stone surfaces (33) and the elevation difference between stone and reference (which gives a direct measure of erosion).

Finally, the relationships between the analytical data obtained and the experimental conditions of the laboratory simulations (current velocity, sediment grain size, test durations, and material properties) were explored.

### Risk Assessment Development.

The general risk assessment for underwater stone heritage was developed in four steps.

1) Selection of a reference model for current velocity vs. time.
  Only few studies addressing the upper-ocean response to extreme weather events show complete temporal trends of current velocity (mostly for tropical cyclones) (44, 46–48, 76–79). Different mooring and drifter datasets were reviewed, selecting the former as they better capture the impact of ocean currents on stationary targets and the typical near-inertial oscillations related to direction changes. To address the effects of a single-direction flow on a stone surface with a specific orientation, Zedler et al. (44)’s simulations were used as a reference; in particular, the velocity evolution of the meridional current component modeled from data collected at 25 m depth during a category 4 hurricane in the N Atlantic. The velocity values were normalized and the same-sign oscillations filtered, obtaining a simplified one-directional reference trend of current velocity, which includes both the storm’s forced stage and the e-folding time of the relaxation stage (about 8 d in total).
2) Creation of calibrated stone decay trends.
  Keeping the same time frame and original temporal evolution, the reference model was linearly rescaled to different peak velocities, namely 0.8, 1.2, 1.5, 1.8, 2.2, and 2.5 m/s, which are the typical maximum current velocities generated by tropical storms and category 1, 2, 3, 4, and 5 tropical cyclones in the SSHWS, respectively (48); since currents are affected by storm intensity and also translation speed, the expected maximum current velocity can be also adjusted using Chang et al. (48)’s equations (it is worth noting that a fast-moving category 5 tropical cyclone may produce virtually the same peak current velocity as a slow-moving category 1 cyclone). The rescaled models were used for converting current velocity in stone surface erosion rate, based on the analytical and experimental data of the laboratory simulations; ultimately, calibrated temporal trends of stone decay in real settings were produced, accounting for the dynamic characteristics of actual currents and differentiating between different seabeds (with coarse or fine sand) and storm intensities.
3) Generation of spatiotemporal data of stone surface erosion.

The calibrated stone decay trends were combined with return period data of storms of diverse genesis and intensity, in order to assess the risk under present and future climate conditions, computed for 100 y but scalable to any time frame. Considering return periods of storms of different strengths allows applying a probabilistic approach and addressing how the vulnerability of underwater stone heritage in specific locations changes over time. The critical storm parameter considered or derived is the 1-min-average maximum sustained wind speed at 10 m.

For tropical storms and cyclones, Bloemendaal et al. (30)’s simulated return periods under present/recent past (1980–2017) and future (2015–2050, IPCC SSP5-8.5 scenario) climate conditions were used. The authors’ approach relies on the use of the statistical model STORM (Synthetic Tropical cyclOne geneRation Model). They first extracted storm statistics from four high-resolution GCMs (General Circulation Models), calculated the projected future changes, and applied these changes to a common baseline of historical observations; then, STORM resampled the four adjusted datasets to generate 10,000 y of synthetic tropical cyclones; finally, by converting the synthetic tracks into 2D wind fields, the authors computed high-resolution wind-speed return periods (this workflow addresses the GCM limitations in capturing local-scale storm behavior due to limited spatial resolution, short simulation periods, and the underrepresentation of the most intense events). The return period trends are shown for areas within a 100-km radius from three coastal locations in each of the ocean basins considered: NW Atlantic, N and SW Indian, and NE, NW, and SW Pacific. For this study, data preprocessing was performed for aggregating the four synthetic datasets used for future simulations, and for calculating the threshold return period, frequency, and probability of occurrence of tropical storms and cyclones for every SSHWS category. Finally, the storm intensity and frequency data were combined with the calibrated stone decay trends to calculate the cumulative material loss of underwater stone surfaces over a 100-y period; the calculations incorporate the variables of climate scenario, proportional contribution of storm classes, stone properties, seabed sediment, and geographic location. The data from each ocean basin were eventually aggregated for clarity in presentation and discussion.

The same approach was also considered for application to other storm types. Extratropical cyclones were eventually excluded, since even the strongest events are typically comparable to SSHWS tropical storms (80–82)—thus, the resulting currents are expected to cause negligible damage to stone surfaces. On the other hand, Mediterranean cyclones (medicanes), although rarer, smaller in scale, and shorter in duration, can be more often associated with stronger winds. For medicanes, Romero and Emanuel (26)’s simulations served as a reference, with return periods under present/recent past (1986–2005) and future (2081–2100, IPCC RCP8.5) climate conditions. The authors’ approach is based on CMIP5 global climate models: They first extracted daily storm-related variables from historical and future simulations; then, large ensembles of candidate synthetic storm tracks were generated using a statistical-deterministic method and submitted to a numerical intensity model; finally, the successful tracks were normalized to observed storm frequencies. Despite being more dated, this study offers the closest correspondence with Bloemendaal et al. (30), and the data processing could be conducted in the same way, referring to equivalent SSHWS ranks.

Regarding overall uncertainty, tropical cyclone frequency and intensity projections show GCM inter-model variability (30), with the IPCC assigning low confidence to long-term frequency changes but high confidence to increases in the proportion of the most extreme events (29). For medicanes, divergent CMIP5 trends exist, but analyses focus on multimodel mean changes and highlight robust patterns based on model consensus, privileging consistency with historical reanalyses (26); however, the authors show the return period trends for the whole Mediterranean basin (the only data of this type available in the climate change literature), thus leading here to an overestimation of the damage calculated for individual sites.

4) Compilation of a shelf sediment map.

To support data visualization, a world seabed sediment map of continental shelf areas was created with the software QGIS 3.40.1-Bratislava. First, the bathymetric map from NOAA’s ETOPO Global Relief Model (83) was used as a base layer and filtered for including only areas with a maximum depth of 200 m; this depth is associated with detectable storm-driven currents (48) and the highest concentration of underwater cultural heritage, and it roughly corresponds to the upper ocean over continental shelf regions. Second, Shom’s global data of seabed sediment grain size (84) were overlaid within the filtered shelf areas, redistributing the original grain-size subdivisions into the main classes of mud, gravel, and sand—with two sand classes, very fine to medium and coarse to very coarse, corresponding to the two sediment types used in the laboratory simulations.

## Supplementary Material

Appendix 01 (PDF)

Dataset S01 (XLSX)

Dataset S02 (XLSX)

Dataset S03 (XLSX)

Dataset S04 (XLSX)

## Data Availability

Study data are included in the article and/or supporting information.
