# Supplementary material for: Underwater cultural heritage and extreme events: Storm impacts under climate change
Source: Proc Natl Acad Sci U S A. 2026 Mar 16;123(12):e2523844123. doi: 10.1073/pnas.2523844123 (PMC13012099; doi:10.1073/pnas.2523844123)
Supplement: Supplementary file 1 — Appendix 01 (PDF) [file pnas.2523844123.sapp.pdf]

PNAS

Supporting Information for
Underwater cultural heritage and extreme events: Storm impacts under climate change

Luigi Germinario, Stuart J. McLelland, Claudio Mazzoli

Corresponding author: Luigi Germinario
Email: luigi.germinario@gmail.com

This PDF file includes:

Figures S1 to S3
Legends for Datasets S1 to S4

Other supporting materials for this manuscript include the following:

Datasets S1 to S4

	Classification & composition	Technical properties	Examples of historical use
MARBLE<br><br>Carrara marble<br>Carrara–Fantiscritti<br>(NW Italy)	Crystalline marble with polygonal to interlobate granoblastic texture and inter-crystalline porosity. Grain size is in the order of hundreds of µm.<br><br>Calcite ≈ 100% | CaO = 97.99% | LOI = 42.40%	Strong with very low porosity<br><br>φ = 0.59%<br>ρ_b = 2.71 g/cm^3<br>ρ_m = 2.72 g/cm^3<br>UCS = 130 MPa	Renowned applications throughout history and worldwide (e.g., the Pantheon of Rome from the Roman Age, the medieval Florence Cathedral complex, Michelangelo's Pietà and David from the Renaissance).
	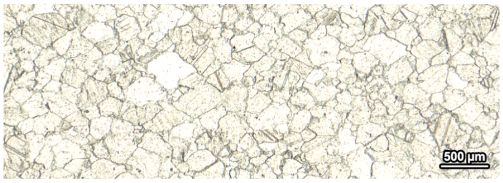	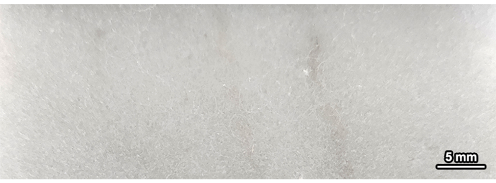	
TRAVERTINE<br><br>Roman travertine<br>Rome–Tivoli<br>(Central Italy)	Boundstone/biolithite with laminations of mixed sparite/clotted peloidal micrite, frequent peloids, rare bioclasts (ostracods), with fenestral, shelter, and mouldic porosity. The most recurring microstructures are shrubs, rafts, reeds, and coated gas bubbles. Grain size ranges from micritic to the order of a few hundred µm.<br><br>Calcite ≈ 100% | CaO = 98.63% | LOI = 44.20%	Strong with medium porosity<br><br>φ = 6.51%<br>ρ_b = 2.50 g/cm^3<br>ρ_m = 2.68 g/cm^3<br>UCS = 110 MPa	Iconic architecture of the city of Rome from the Roman Age (e.g., the Colosseum, the Theater of Marcellus, the Roman Forum) and Baroque (Trevi Fountain, the Spanish Steps, Bernini's works in St. Peter's and Navona squares).
	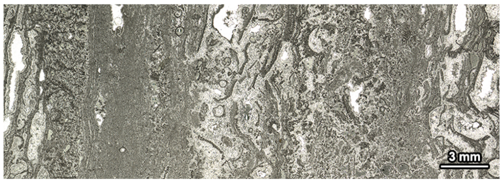	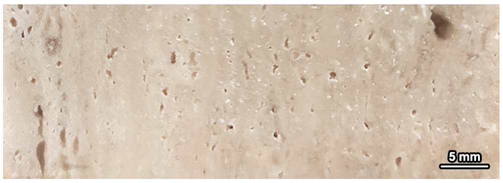	
COMPACT<br>LIMESTONE<br><br>Istria stone<br>Vrsar/Orsera<br>(W Croatia)	Stylolitic mudstone/micrite, with sporadic sparite and allochems (peloids and bivalves) and intercrystalline and fracture porosity. Grain size is mostly micritic (<5 µm).<br><br>Calcite ≈ 100% | CaO = 98.44% | LOI = 42.86%	Very strong with very low porosity<br><br>φ = 0.56%<br>ρ_b = 2.70 g/cm^3<br>ρ_m = 2.71 g/cm^3<br>UCS = 220 MPa	Prestigious buildings of the city of Venice erected from the Middle Ages (e.g., Doge's Palace), through the Renaissance (Rialto Bridge, Redentore Church) and Baroque (the Bridge of Sighs, La Salute Church).
	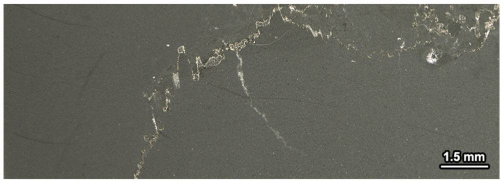	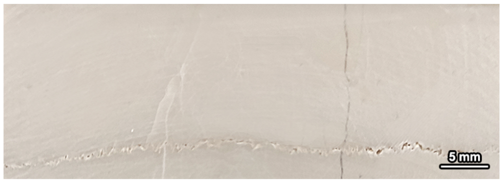	
POROUS<br>LIMESTONE<br><br>Vicenza stone<br>Vicenza–Costozza<br>(NE Italy)	Grainstone/biomicrite made of coralline algae, foraminifers (mainly nummulites and miliolids), and echinoderms, with minor bryozoans and mollusks (gastropods and bivalves). Porosity is inter- and intraparticle. Crystal size is a few tens of µm to micritic, but bioclasts may have a millimetric size.<br><br>Calcite ≈ 100% | CaO = 98.89% | LOI = 43.06%	Soft with high porosity<br><br>φ = 28.74%<br>ρ_b = 1.93 g/cm^3<br>ρ_m = 2.71 g/cm^3<br>UCS = 28 MPa	Acclaimed works by the Renaissance architect Palladio, especially in the city of Vicenza (e.g., the Palladian villas, Basilica Palladiana, the Olympic Theater).
	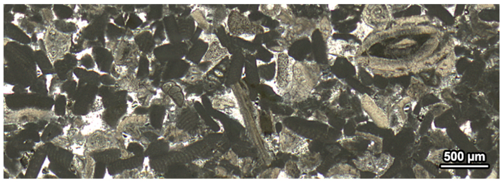	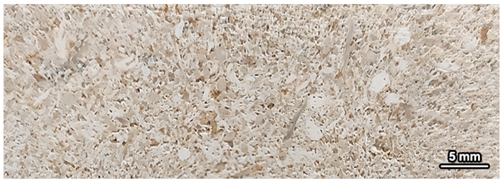	

Fig. S1. Petrographic, geochemical, and technical properties of the stone materials and their historical usage, with thin-section photomicrographs in plane-polarized light and macrophotographs (φ = open porosity; ρ_b & ρ_m = bulk & matrix density; UCS = uniaxial compressive strength; the complete XRF dataset is in Dataset S4) (figure and data from 18).

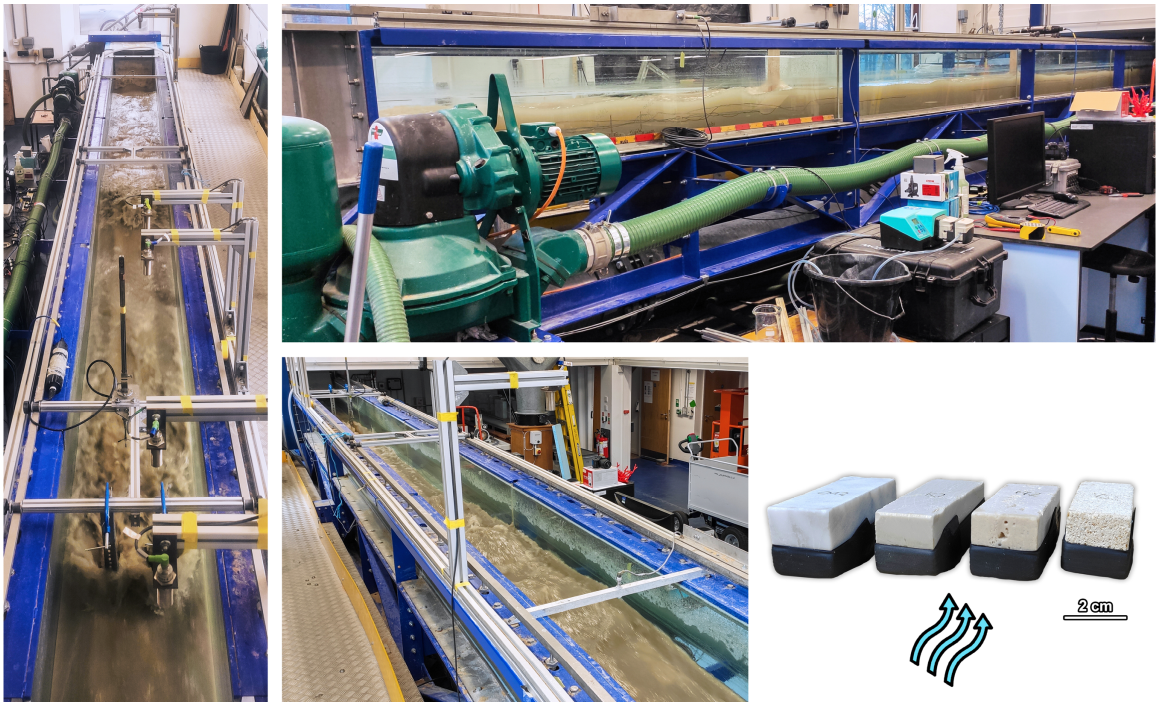

Fig. S2. The flume during operation. Four stone specimens are also shown, with their exposed (top) and shielded (bottom) surfaces.

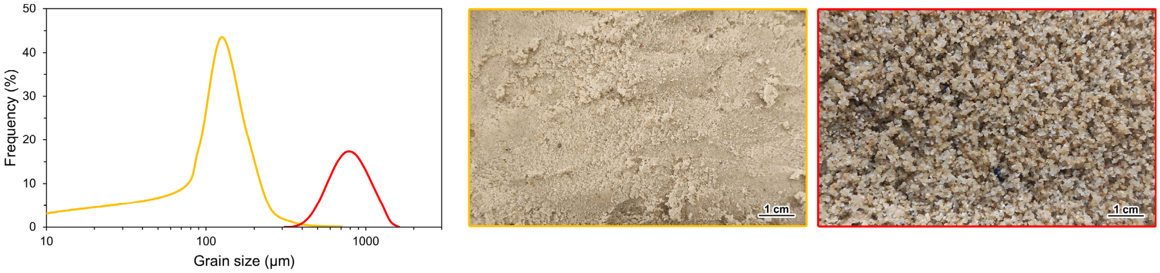

Fig. S3. Grain size distributions of the fine (in yellow) and coarse (in red) sands used for the flume experiments.

Dataset S1 (separate file). Full dataset of material loss and textural alteration measured on the 3D models created by optical profilometry for all stone specimens tested in the flume with different flow velocities and sediment sizes.

Dataset S2 (separate file). Stone material loss calculated for single storms of different intensities and for different seabed sediment sizes, combining the reference model of current velocity by Zedler et al. (44), the peak velocities provided by Chang et al. (48), and the experimental data of the flume simulations.

Dataset S3 (separate file). Stone material loss calculated over a 100-year period for different locations, storm intensities, and seabed sediment sizes, under present and future climate scenarios, using the storm return period data provided by Romero & Emanuel (26) and Bloemendaal et al. (30).

Dataset S4 (separate file). Full chemical composition by XRF of the four stone materials investigated (from 18).
